# Microsurgical resection of an inferior cerebellar peduncle cavernous malformation: 3-Dimensional operative video

**DOI:** 10.3171/2019.7.FocusVid.19147

**Published:** 2019-07-01

**Authors:** Guilherme H. W. Ceccato, Rodolfo F. M. da Rocha, Julia Goginski, Pedro H. A. da Silva, Gabriel S. de Fraga, Marcio S. Rassi, Luis A. B. Borba

**Affiliations:** 1 School of Medicine, Federal University of Paraná, Curitiba, PR, Brazil;; 2 School of Medicine, Faculdade Evangélica do Paraná, Curitiba, PR, Brazil;; 3 School of Medicine, Faculdades Pequeno Príncipe, Curitiba, PR, Brazil;; 4 Department of Neurosurgery, Evangelic University Hospital of Curitiba, PR, Brazil;; 5 Department of Neurosurgery, Federal University of Paraná, Curitiba, PR, Brazil

**Keywords:** brainstem, inferior cerebellar peduncle, cavernous malformation, cavernoma, video

## Abstract

Brainstem cavernous malformations are especially difficult to treat because of their deep location and intimate relation with eloquent structures. This is the case of a 26-year-old female presenting with dizziness, dysmetria, nystagmus and unbalance. Imaging depicted a lesion highly suggestive of a cavernous malformation in the left inferior cerebellar peduncle. Following a suboccipital midline craniotomy, the cerebellomedullary fissure was dissected and the lesion was identified bulging the surface. The malformation was completely removed with constant intraoperative neurophysiological monitoring. The patient presented improvement of initial symptoms with no new deficits. Surgical resection of brainstem cavernous malformations can be successfully performed, especially when superficial, using the inferior cerebellar peduncle as an entry zone.

The video can be found here: https://youtu.be/-GGZe_CaZnQ.

## Transcript

This is a 3-dimensional operative video of the microsurgical resection of an inferior cerebellar peduncle cavernous malformation. The patient was a 26-year-old female presenting with a two weeks history of dizziness, dysmetria, nystagmus and unbalance.

### 0:36 Preoperative imaging

Preoperative MRI demonstrated a mass in the left inferior cerebellar peduncle, with mixed signal in T1 and T2 enhancing heterogeneously after contrast administration and with blooming artifact in T2 star weighted image, suggestive of a cavernous malformation. (Batra et al, 2009) Here we see the location of the lesion in the inferior cerebellar peduncle and a bulging area in the floor of the fourth ventricle.

### 1:05 3D models

In this 3D model we can better understand the positioning of the lesion to be approached. Because of progressive worsening of symptoms and the lesion was located superficially, microsurgical excision was indicated. (Giliberto et al, 2010; Xie et al, 2018) A suboccipital midline approach was performed. We note that the malformation is hidden by cerebellum, but could be reached through a route below it, dissecting the region of cerebellomedullary fissure. (Asaad et al, 2010; Ferguson et al, 2018; Giliberto et al, 2010; Lawton et al, 2006; Mussi and Rhoton, 2000)

### 1:37 Procedure

Initially the cisterna magna is opened, and also the lower half of the roof of the fourth ventricle. (Mussi and Rhoton, 2000) The cerebellomedullary fissure is dissected and we see a segment of the vertebral artery. An ipsilateral PICA segment is dissected and detached. We see a bulging area in the inferior cerebellar peduncle, with surface discoloration. With bipolar forceps this area is coagulated and opened, giving access to the lesion. (Cavalcanti et al, 2016; Deshmukh et al, 2014) Recent blood is identified, and gentle combination of suction and traction is employed during resection. We see yellowish parts, consistent with hemosiderin. Then progressively the lesion is carefully detached from the brainstem. Intraoperative monitoring is used and helps to guide resection. (Asaad et al, 2010; Giliberto et al, 2010) Then the malformation is completely removed en bloc…with a minimal remaining part removed in sequence. The cavity is inspected about remaining lesion, and regarding hemostasis. In this view we observe the site approached, near the vagal and hypoglossal trigones in the inferior part of the floor of fourth ventricle. (Mussi and Rhoton, 2000)

Here we review some pictures of the procedure, since the opening of cisterna magna, dissecting the region of cerebellomedullary fissure and detaching the PICA from the tonsil, and starting to expose the bulging area in the inferior cerebellar peduncle; (Mussi and Rhoton, 2000) we see the site that will be approached with surface discoloration and here observe the surgical cavity.

### 3:29 Postoperative imaging

Postoperative MRI demonstrated complete resection

### 3:37 Outcome

Pathology confirmed the lesion to be a cavernous malformation. The patient presented improvement of symptoms, with no new neurological deficits in the follow-up.

Here we demonstrate preservation of tongue motility, with no deficit of the hypoglossal nerve, and preservation of swallowing, with no damage to the vagus nerve, whose nuclei were closely related to the region approached in the brainstem.
